# Integrated Lateral Flow Device for Flow Control with Blood Separation and Biosensing

**DOI:** 10.3390/mi8120367

**Published:** 2017-12-20

**Authors:** Veronica Betancur, Jianbo Sun, Nianqiang Wu, Yuxin Liu

**Affiliations:** 1 Department of Mechanical and Aerospace Engineering, West Virginia University, Morgantown, WV 26506, USA; vbetancu@mix.wvu.edu (V.B.); nick.wu@mail.wvu.edu (N.W.); 2 Lane Department of Computer Science and Electrical Engineering, West Virginia University, Morgantown, WV 26506, USA; jnsun@mix.wvu.edu

**Keywords:** lateral flow device, polydimethylsiloxane (PDMS) surface modification, flow control, integration of functions

## Abstract

Lateral flow devices are versatile and serve a wide variety of purposes, including medical, agricultural, environmental, and military applications. Yet, the most promising opportunities of these devices for diagnosis might reside in point-of-care (POC) applications. Disposable paper-based lateral flow strips have been of particular interest, because they utilize low-cost materials and do not require expensive fabrication instruments. However, there are constraints on tuning flow rates and immunoassays functionalization in papers, as well as technical challenges in sensors’ integration and concentration units for low-abundant molecular detection. In the present work, we demonstrated an integrated lateral flow device that applied the capillary forces with functionalized polymer-based microfluidics as a strategy to realize a portable, simplified, and self-powered lateral flow device (LFD). The polydimethylsiloxane (PDMS) surface was rendered hydrophilic via functionalization with different concentrations of Pluronic F127. Controlled flow is a key variable for immunoassay-based applications for providing enough time for protein binding to antibodies. The flow rate of the integrated LFD was regulated by the combination of multiple factors, including Pluronic F127 functionalized surface properties and surface treatments of microchannels, resistance of the integrated flow resistor, the dimensions of the microstructures and the spacing between them in the capillary pump, the contact angles, and viscosity of the fluids. Various plasma flow rates were regulated and achieved in the whole device. The LFD combined the ability to separate high quality plasma from human whole blood by using a highly asymmetric plasma separation membrane, and created controlled and steady fluid flow using capillary forces produced by the interfacial tensions. Biomarker immunoglobulin G (IgG) detection from plasma was demonstrated with a graphene nanoelectronic sensor integrated with the LFD. The developed LFD can be used as a flexible and versatile platform, and has the potential for detecting circulating biomarkers from whole blood. Sandwich-immunoassays can be performed directly on the LFD by patterning receptors for analytes on a desired substrate, and detections can be performed using a variety of sensing methods including nanoelectronic, colorimetric, or fluorescence sensors. The described bio-sensing technology presents an alternative for POC testing using small samples of human whole blood. It could benefit regions with limited access to healthcare, where delays in diagnosis can lead to quick deterioration of the quality of life and increase the morbidity and mortality.

## 1. Introduction

The second half of the 20th century has brought great advances in biochemistry and technology, leading to greater sensitivity and more specific analysis of samples [1]. A manifestation of this potential is in the emergence of microfluidic bioanalysis, which concerns the controlled transport of low volumes of fluid, and it is the key for miniaturization, as well as the integration of multiple functionalities, allowing for the performance of complete laboratory protocols on a single chip [2]. Performing analytical and diagnostic tasks in microfluidic-based systems presents several advantages, including small sample volume requirements, rapid transport time and portability, as well as allowing for continuous flow with high precision, greatly improving the efficiency and reliability of devices for point-of-care (POC) diagnostics, which could help in bridging the healthcare gap between developed and developing countries [3,4,5,6]. 

While the development of microfluidic technology for diagnostics and disease monitoring has evolved rapidly over the past years, to date, these technologies still face several challenges for becoming truly viable for point-of-care testing (POCT) [7]. These limitations include the lack of integration of all function units, handling complexity, sample treatment, and requirement of large and costly external equipment for flow initiation and control as well as detection, which are all great limitations especially for low-resource settings [3,8,9,10]. Integrated microfluidic devices could offer a higher degree of accuracy, contamination control, ease of use, rapid results, and low cost [11]. To date, only a small number of devices offer total analysis on chip, including reagent storage, and sample collection and preparation, or the ability to test crude real-world samples (e.g., blood, urine, and saliva) [12,13,14,15]. 

In addition, the applications of such systems in home and POC situations are still limited, because they typically require external macroscopic actuators, bulky fluidic connections, and electromechanical interfaces to initiate and control fluid flow. Passive flow methods are alternatives in overcoming these limitations, because they demand no external power input, which makes them quite compact and thus portable, resulting in attractive analytical tools for applications, such as single use assays and simple diagnostics [3,5]. Capillary based pumping takes advantage of surface tension to pull fluid through the device, and the spontaneous fillings of the microfluidic network are functions of the device’s design, and surface properties of internal channels. The pioneer work for capillary pumping systems had been demontrated by Delamarche et al. They characterized the effects of dimensions of microstructures and their spacings on the progression rates of a liquid in both vertical and horizontal directions in the capillary systems [6,10]. Their capillary pumping systems were made in silicon coated with a thin gold film, to further assist fluid flow. However, the silicon and deposited thin gold film are expensive, and the fabrication processes require cleanroom facilities. Naturally hydrophilic polymers (such as NOA-63) have been reported for the fabrication of self-powered chips [16]. The cured NOA-63 layer is rigid, and there could be some limitations in device integration and channel bonding and sealing. Disposable paper-based lateral flow strips have been most commonly applied for immunoanalysis because of low-cost materials and easy fabrication [17,18], however, they are normally opreated in an open channel mode, and there are constraints on tuning flow rates and technical challenges in sensors’ integration. 

The research work in this paper demonstrated an integrated passive microfluidic lateral flow device (LFD). This device combines the ability to perform plasma separation from human whole blood, control the flow rate, integrate the detection sensor, and perform biomarker sensing, which shortens the time of sample processing, as well as allowing for rapid detection. The flow in the LFD is powered by the capillary force, which was accomplished by two means: the use of capillary pumps (CPs) and the surface functionalization of polydimethylsiloxane (PDMS) using Pluronic F127, thus eliminating the need of external power and reducing the cost. Pluronic copolymers have extremely low toxicity and immunogenic response, and are widely used in many biotechnological applications [19]. They are approved for medical applications by the US Food and Drug administration [20]. The molecules of Pluronic attached to the hydrophobic polymer (PDMS) can effectively repel proteins [20]. The modified PDMS surface is hydrophilic, and can significantly reduce non-specific adsorption of proteins [21,22,23]. In our work, detailed studies for controlling flow rates were performed in regarding to the surface characteristics of PDMS functionalized with different Pluronic concentrations, surface treatments with/without water, and by varying flow resistance of the integrated flow resistor. The promise of this novel LFD lies in its potential for POC applications, offering an integrated and flexible platform, which can perform laboratory procedures in one single chip.

## 2. Materials and Methods

### 2.1. Integrated Lateral Flow Device Design

**Figure 1:**
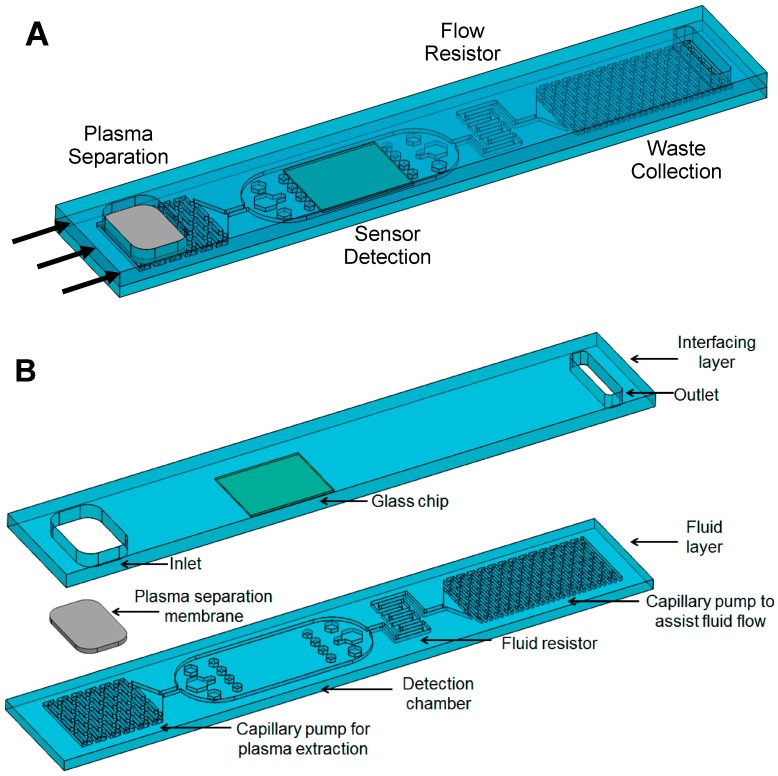

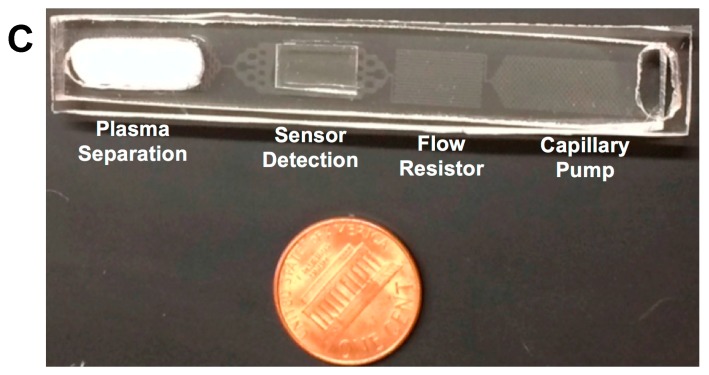
(**A**) Schematic diagram of an assembled lateral flow device (LFD) for detection of single biomarker in whole blood sample. The black arrows indicate the flow direction; (**B**) Schematic diagram shows interfacing layer and fluid layer of the LFD. The first capillary pump (CP1) is partially covered by a plasma separation membrane, and it is designed for plasma extraction, while the second capillary pump (CP2) is designed to assist the fluid flow. Surface-immunoassays can be performed directly on the sealed chip on top of the detection chamber area; (**C**) A picture shows the fabricated LFD with the same design in Figure 1A.

A LFD operated under the capillary force was designed to integrate several functional units, including plasma separation, capillary pumps for fluid transport, flow resistor, and embeded on-chip sensor detection. As shown in Figure 1, the integrated LFD is composed of two layers, a bottom fluid layer and a top interfacing layer. The fluidic layer consists of a membrane-based plasma separation unit, a capillary pump (CP) for plasma extraction/delivery unit, a detection chamber, a flow resistor, and a second CP for assisting fluid flow and waste collection. The interfacing layer consists of the reservoir for whole blood loading, an embedded pre-functionalized sensor substrate, and waste collection. The plasma separation membrane needs to be trimmed and put in a close contact with the underneath microstructures in the plasma separation CP. Fluid dividers were designed to uniformally distribute fluid flowing from a narrow channel (connected to the plasma separation) to a wider detection chamber, as shown in Figure 1. 

Ideally, the CPs should generate smooth flow of the fluid, filling the CP completely without entrapping air. Therefore, the CP must have low flow resistance and high capillary pressure. Advanced capillary pumping structures can further enhance the pumping capabilities [10]. In our LFD, the CP for plasma extraction was designed with symmetric elongated structures (260 μm wide, 1041 μm long, and with a 260 μm spacing between the microstructures) to control the filling front of the fluid, and the CP for assisting fluid flow and waste collection was designed with symmetric elongated microstructures as well (160 μm wide, 640 μm long, and with a 160 μm spacing between the microstructures). In CPs with symmetric structures, the resulting filling front is parallel to the microstructures. For an enclosed CP, such as ours, there is a risk of entrapping air; symmetric structures reduce this risk because the filling fronts of liquid converge from the edges of the CP towards its center [10].

Because the binding reaction between the antigen and the detection and capture antibodies takes a determined amount of time to reach equilibrium, accurate control of the flow is the key to provide enough time for protein binding to antibodies and biomarker detection. Fluidic resistance of microchannels allow for modulating of the flow rate passively [24]. In our case, the flow resistor was designed as periodic serpentine channels with a rectangular cross-section. The flow resistance is determined by the dimensions of the flow resistor. Here, the width and depth of the flow resistor channel were kept constant at 200 µm and approximately 20 µm, respectively, and the resistance of fluid resistor was modified by means of varying the length of the flow resistor, which depends on the number of serpentine channels. 

### 2.2. Fabrication

Materials dictate the properties of the microfluidic flow path to a large extent. They influence flow rate, capillary pressure, wetting, optical properties, adhesion of biomolecules, cost, and the fabrication methods [1]. 

PDMS is a clear, colorless silicon-based organic polymer, and it is the most popular material for microfluidic research and prototyping, due to its ease of fabrication, biocompatibility, nontoxicity, optical transparency, and gas permeability. However, because of its hydrophobic nature and strong adsorption of biological molecules, such as proteins, PDMS is not suitable to be directly used to fabricate devices used in analytical, biological, and clinical applications. In this work, PDMS (Sylgard 184) was functionalized with Pluronic F127 (Sigma-Aldrich Co., St. Louis, MO, USA) to modify its hydrophobic surface for LFD fabrication and operation. The molecules of Pluronic have a triblock structure, consisting of one hydrophobic polypropylene oxide (PPO) group accompanied by two hydrophilic polyethylene oxide (PEO) units [22]. The general structure is PEO*_n_*–PPO*_m_*–PEO*_n_*, where *m* and *n* vary based on the type of Pluronic (for Pluronic F127, *m* = 100, *n* = 65, molecular weight = 12,600) [21,22]. The PPO chain attaches to the hydrophobic polymer (PDMS), while the PEO chains self-assemble into a cilia-like surface on the PDMS, effectively repelling proteins [20]. To prepare the Pluronic/PDMS mixture, 200 mg of Pluronic F127 were dissolved in 1 mL of alcohol. Samples with 20 µL (2 µL/g) and 40 µL (4 µL/g) of this complex were mixed thoroughly with the freshly made uncured PDMS (10 g). The mixture was then ready for the device fabrication. The Pluronic/PDMS samples of 2 µL/g were as clear as the unmodified PDMS, and the samples with concentrations higher than 4 µL/g showed different levels of cloudiness. To study and compare the fluid behaviors, two concentrations of Pluronic (2 µL/g and 4 µL/g) were used to functionalize PDMS in this work.

The fluid layer (Figure 1B) was fabricated using standard photolithography and soft lithography techniques. The process consisted of utilizing a high-resolution photomask to expose and polymerize a polymer-based photoresist spun cast onto a silicon wafer. Following development, the resulting pattern was used as a master mold for replication in Pluronic F127 functionalized PDMS. In details, SU-8 2025 negative photoresist (Microchem, Westborough, MA, USA) was used for fabricating the fluidic layer mold. SU-8 2025 was spin coated on a silicon wafer to a thickness of 20 μm, exposed to UV radiation, and rinsed in SU-8 developing solution, to create the microchannel templates. Channel height was then measured using a Stylus Profilometer. The Pluronic/PDMS mixed solution was then cast over the SU-8 master mold, creating an inverse replica of the design, and cured for one hour at 60 °C. Once cured, the Pluronic/PDMS layer was removed from the master mold.

The interfacing layer (Figure 1B) was fabricated by casting the Pluronic/PDMS mixture over the embedded sensor substrate. For example, the substrate first needs to be trimmed to a size comparable to the detection chamber, and the interfacing layer was then fabricated by placing the chip on a Petri dish, and casting the Pluronic functionalized PDMS on the dish, followed by degassing and curing for 1 h at 60 °C. This resulted in a clean and flat Pluronic functionalized PDMS surface, with the substrate embedded in it. 

An inlet and an outlet for the fluid were cut out from the interfacing layer using a sharpened puncher. In an aqueous environment, Pluronic molecules migrate towards the surface, due to the high gradient between the water and the PDMS substrate, resulting in a reduction of non-specific adsorption and an enhancement of wetting [21,22]. For this reason, both layers were immersed in de-ionized water for 24 h at room temperature. After the 24 h immersion, the surfaces of both the flow and the interfacing Pluronic functionalized PDMS layers were dried with nitrogen gas, and then exposed to oxygen plasma treatment for 30 s. The flow layer was immediately placed in conformal contact with the interfacing layer, irreversibly bonding them together, ensuring that the sensor substrate on the interfacing layer was placed directly on top of the detection chamber on the flow layer, and then stabilized in an oven at 60 °C for half an hour. A fabricated microfluidic LFD used for flow control tests was shown in Figure 1C. Non-water treated devices were also fabricated and used to compare with the water treated ones for flow control tests. 

### 2.3. Pumping Mechanism and Flow Rate Control

Flow control is a key in the proper functioning of microfluidic devices, and it can be achieved by using a combination of valves, pumps, and flow resistances [1]. The overall flow rate of the fluid is encoded by the pumping mechanism, which can be either active (e.g., reciprocating movement membrane valve, capillary electrophoresis using electro-osmotic flow, centrifugal force in lab on a CD devices, electrowetting) or passive (e.g., porous capillary membrane, capillary pump, vacuum driven suction) depending on the application requirements [1,25].

Passive microfluidic systems, a function for the design, have spontaneous filling, and present an attractive alternative, due to their portability, zero power consumption, low volume requirement, low cost, and ease of handling [1].

#### 2.3.1. Dynamics of Capillary Flow in a Microchannel

In autonomous capillary systems, liquids are displaced by means of capillarity, and their proper functioning requires displacing accurate volumes of liquids with precise flow rates [10]. The flow velocity of a liquid in a conduit can be derived from solving the Navier−Stokes equations. The flow rate of a liquid is a function of viscosity of the fluid, capillary pressure, and total flow resistance of the flow path (governed by the device’s geometry), and can be calculated by integrating the flow velocity (Equation (1)) [2].

(1) $$Q=\frac{\Delta P}{8\eta }\times \frac{A\times R_{H}}{L}$$

where *η* is the viscosity of the fluid, Δ*P* is the differential pressure between the inlet and the outlet, *L* is the length, *A* is the area, and *R_H_* is the hydraulic radius of the microchannel. During filling by capillary action, Δ*P* can be replaced with the pressure generated at the liquid meniscus, or capillary pressure (*P_c_*) [2,10]. For the purpose of our study, the viscosity of plasma was measured using a viscometer at room temperature, and the value was found to be 1.66 mPa∙s. The measured value for the viscosity of human blood plasma is comparable to the reported one that was measured at room temperature [26].

The geometric and constant parameters can be regrouped for convenience into a unique parameter that we call the total flow-rate resistance (*R_F_*), resulting in a simplified equation for flow rate (Equation (2)) [2].

(2) $$Q=\frac{1}{\eta }\times \frac{\Delta P}{R_{F}}=\frac{1}{\eta }\times \frac{P_{c}}{R_{F}}$$

The *R_F_* of the flow path for a rectangular microchannel is not trivial to calculate, but it can be approximated by a linear term, as long as the microchannel depth (*a*) is much smaller than the microchannel width (*b*) (Equation (3)) [2,27].

(3) $$R_{F}={\left[\frac{1}{12}\left(1+\frac{5a}{6b}\right)\times \frac{abR_{H}^{2}}{L}\right]}^{-1}$$

(4) $$R_{H}=\frac{2A}{p}=\frac{ab}{a+b}$$

When filling the microchannel, due to capillary action, the parameter *L* corresponds to the filled length of the channel; consequently, it increases with the advancement of the filling front of the liquid in the channel. For a system with varying cross-sections connected in series, the total *R_F_* of the system can be calculated as the sum of the different components with constant cross-sections [2].

#### 2.3.2. Capillary Pressure

The capillary pressure (*P_c_*) is a function of the depth and width of the microchannels, the surface tension (*γ*), and contact angles on the bottom, top, left, and right side walls [1]. The capillary resistance is a function of the geometry of the channel, and together with capillary pressure and viscosity of the fluid (*η*), it can aid in calculating the flow rate [1]. 

The *P_c_* is the difference in pressure across the interface between two immiscible fluids, in this case plasma and air, and can be described by the Young–Laplace equation. For a microchannel with a rectangular cross section, the curvature along the depth and width axes contributes to the meniscus pressure [2]. *P_c_* can then be calculated using Equation (5) [2,28].

(5) $$P_{c}=-\gamma \left(\frac{cos\alpha_{b}+cos\alpha_{t}}{a}+\frac{cos\alpha_{l}+cos\alpha_{r}}{b}\right)$$

where *γ* is the surface tension of liquid, and *α_b_*_,*t*,*l*,*r*_ are the contact angles of the liquid on the bottom, top, left, and right wall of the microchannel, respectively. 

When using the static contact angles to calculate *P_c_*, the four contact angles are assumed to be equal in magnitude, simplifying the calculation (Equations (6)–(8)).

(6) $$P_{c}=\gamma \left(\frac{1}{R_{Height}}+\frac{1}{R_{Width}}\right)$$

(7) $$R_{Height}=\frac{a}{2cos\alpha }$$

(8) $$R_{Width}=\frac{b}{2cos\alpha }$$

where *R_Height_* and *R_Width_* are the minor and major radii of curvature respectively, and *α* is the static contact angle.

In this work, the dynamic contact angles were also considered, measured, and further analyzed for flow control, because the dynamic contact angles of the liquid with the walls of the microchannel depend on the speed of flow, as well as the receding or advancing state of the interface [2].

#### 2.3.3. Contact Angle Measurement

Because contact angles on the channel wall surfaces may be significantly different from smooth and flat surfaces [29], both the static and in situ dynamic contact angles were measured and compared, to study the wettability of PDMS surfaces with/without Pluronic functionalization to understand the effects of resistance variation and Pluronic concentration in the PDMS, and verify the hydrophilicity of the surface. Static contact angle on the flat surface was measured using a handheld digital microscope (Celestron LLC, Torrance, CA, USA), by placing the microscope inclined about 15–20 degrees, such that an image of the droplet outline and its reflection on the substrate can be taken. Static contact angles were analyzed using Image J software. For the dynamic contact angle measurement, a drop of liquid (40 µL) was placed in the inlet port of the LFD, and the air–liquid interface was recorded by performing a time-lapse imaging using a Nikon Eclipse Ti microscope (Nikon, Tokyo, Japan), with objective lenses for magnification from 4× to 20×. NIS-Elements Viewer (Nikon) was then used for analysis. Experiments were performed for human blood plasma on both water treated and non-water treated Pluronic functionalized PDMS. Concentrations of Pluronic/PDMS mixture (2 µL/g and 4 µL/g) were taken into account. The Pluronic/PDMS concentrations lower than 2 µL/g were also studied, but the contact angle variation was very small, while Pluronic/PDMS concentrations higher than 4 µL/g turned the PDMS layer cloudy, which could conflict with optical detection methods. 

In addition, the dynamic contact angles were measured for all LFDs integrated with different flow resistors, in order to evaluate the relationship between the resistance and contact angle. 

Note that in this work, we describe two types of fluidic resistances. The first, the flow-rate resistance is considered as a function of the geometry of the microchannels alone (Equation (3)), while the second, hydraulic resistance, accounts for not only the geometric parameters, but also the viscosity of the fluid flowing through the channels. The designed flow resistors with different resistances (flow-rate resistance and hydraulic resistance of plasma) were listed in Table 1.

### 2.4. Evaluation of the Integrated LFD with Human Whole Blood

A major requirement for the development of POC tests for detection of disease analytes is the need to separate high-quality plasma from whole blood efficiently and rapidly. Methods such as centrifugation require additional equipment to separate the plasma fraction from red blood cells [30], so they are not convenient for POC applications. The highly asymmetric nature of a plasma separation membrane allows cellular components of the blood (red cells, white cells, and platelets) to be captured in larger pores without lysis, while the plasma flows down into smaller pores on the downstream side of the membrane. Additionally, plasma separation membranes are inexpensive, and easy to integrate in microfluidic platforms.

The blood separation membrane Vivid^TM^ GR (Pall Corporation, Port Washington, NY, USA) was selected for the utilization in the LFD, because it has the highest plasma yield (~80%) according to the manufacturer. After the LFD was assembled, the plasma separation membrane was trimmed to a size slightly smaller than that of the window opening (18 mm by 5 mm), and placed on top of the capillary pump for plasma separation. The contact between the membrane and the microstructures in the capillary pump needs to be very good for a successfully separation. 

Human whole blood with anticoagulant K2 EDTA was purchased from Innovative Research (IPLA-WB1, Novi, MI, USA). The protocol for using human blood was approved by West Virginia University (IBC #15-07-02).

### 2.5. Integrated Nanoelectronic Biosensing 

#### 2.5.1. Fabrication of Graphene-Based Nanoelectronic Biosensor and Integration with LFD

To demonstrate biosensing using the LFD, a graphene field effect transistor (GFET) biosensor was fabricated and integrated with the LFD for detection of immunoglobulin G (IgG). To fabricate the GFET biosensors, chemical vapor deposited graphene was transferred onto glass slide using poly(methyl methacrylate) as the supporting layer followed by thermal annealing and electrolytic cleaning to remove the polymeric residues [31]. Nickel (5 nm) and gold (45 nm) thin films were deposited using e-beam evaporator, and then the gold layer was etched to form the gate, source, and drain electrodes using gold etchant, respectively. After the second photolithography, the nickel layer was etched to be a shielding layer for the patterning of the graphene channel. The graphene channel was then patterned using dedicated oxygen plasma. After removing the remaining nickel layer, another 10 nm nickel layer was deposited to serve as a protective layer during the subsequent integration with the LFD. 

The GFET biosensor chip was embedded in the interfacing layer by casting Pluronic functionalized PDMS on it. After curing, the interfacing layer was peeled off, and the inlet and the outlet were punched. The protective nickel layer was then etched off using nickel etchant. The fluid layer and the interfacing layer were then immersed in deionized water for 24 h, to render the surfaces hydrophilic. Due to high vulnerability of graphene, the interfacing layer cannot be exposed to oxygen plasma. Alternatively, a water layer was left on it to facilitate the bonding. The fluid layer was bonded onto the interfacing layer after being activated in oxygen plasma. The sample was placed in a 60 °C oven for 3 h, to allow complete bonding.

#### 2.5.2. Functionalization of GFET Biosensor

The graphene channel was then functionalized with 5’-amino modified human immunoglobulin G (IgG) aptamer using 1-pyrenebutanoic acid *N*-succinimidyl ester (PBASE) (Sigma-Aldrich) as the linker. Briefly, PBASE (10 mM, dissolved in dimethyl sulfoxide (DMSO)) was injected into the channel, and kept for 2 h. After rinsing with DMSO, 5′-amino modified IgG aptamer (100 μM in 1× PBS (10 mM); Base Pair Biotechnologies, Inc., Pearland, TX, USA) was injected into the channel and incubated for 3 h, to allow the conjugation with PBASE. The remaining unconjugated sites were then blocked by bovine serum albumin (BSA, 10% *w*/*v* in 1× PBS). After functionalization, the channel was rinsed with 1× PBS and kept in 1× PBS in refrigerator until usage. 

#### 2.5.3. Signal Measurement

For the detection of IgG, the electrical measurements were performed using Keithley 4200 SCS. The gate voltage was set to be 0 V, and the source to drain voltage was set to be 0.01 V. The drain current was measured in response to IgG.

### 2.6. Microscope Imaging and Data Analysis

A Nikon Eclipse Ti microscope (Nikon) was used to image and evaluate the flow separation capabilities of the membrane for whole blood separation and the flow speeds. During the imaging, a 40 μL droplet of liquid was placed in the inlet port of the device, and the fluid was transported by capillarity, and a time-lapse recording was taken in the flow resistor area in order to observe the combined effects of flow resistance and Pluronic concentration. For analysis of blood separation efficiency, 30 μL of human whole blood was deposited on the GR plasma separation membrane, and a time-lapse recording was taken in the capillary pump area, to image a contact region of the plasma separation membrane with the capillary pump microstructures.

Flow speed was analyzed at the entrance and middle of the flow resistor, to observe its dynamic change due to the pressure drop between the beginning and end of the rectangular microchannel [32]. The overall flow rate of the device was calculated by timing the fluid from entrance to exit of each component, and then dividing the volume of the specific component by the time that the fluid spent going across it. Blood separation efficiency was analyzed using microscopy techniques to evaluate the quality of the separated plasma. Flow rate and flow speed analysis were performed using the time-lapse results by the NIS-Elements Viewer (Nikon). Dynamic contact angle measurements were performed manually using a protractor. For statistical analysis, data was presented as the mean ± standard deviation (SD) and each individual experiment was performed at least three times (*n* ≥ 3). The results were evaluated by the t test and single factor analysis of variance (ANOVA). 

## 3. Results and Discussions

The LFD was fabricated on PDMS using soft lithography, and sealed with a blank piece of PDMS. Because PDMS is of hydrophobic nature, it suffers serious non-specific protein adsorption, therefore requiring surface modification strategies [12]. Nonionic surfactants can strongly adsorb on hydrophobic surfaces and render them hydrophilic and nonionic, thus preventing the interaction between proteins and the surface [12]; with this in mind, the PDMS surface was functionalized with Pluronic F127 to render it hydrophilic. The concentration of Pluronic was optimized for the sake of controlling the flow rate, and allowing performing reliable biomarker detection. In addition, the Pluronic F127-modified PDMS has been shown to significantly suppress the non-specific adsorption of proteins, due to the improved hydrophilicity, compared to native PDMS [33,34]. 

The effects of surface modifications and variations of the flow resistor’s resistance on the characteristics of the capillary flow were studied in five different microfluidic devices. Table 2 presents a summary of the parameters measured in this work.

### 3.1. Static and Dynamic Contact Angles 

In general, a static contact angle is measured from a smooth flat surface in an open air environment, however, it does not account for the resistance encoded by the constricted microchannel and the microstructures in the capillary pumps, such as their shape, size, and spacing between microstructures within the microfluidic device, and it can greatly differ from an in situ dynamic contact angle. In this study, static contact angles of plasma were measured by dispensing a droplet of liquid on the surface of the Pluronic functionalized PDMS with and without water treatment, respectively. The angles were continuously monitored for a period of 20 min to provide enough time for the droplet contact angle to be stabilized. As a point of comparison, dynamic contact angles were measured using Nikon Eclipse Ti microscope. For the flow resistor, dynamic contact angle measurements were always taken at the entrance channels for consistency. The inverted microscopy technique allowed for measuring dynamic contact angles on the top and bottom channel surfaces (*α_b_* and *α_t_*). 

**Figure 2:**
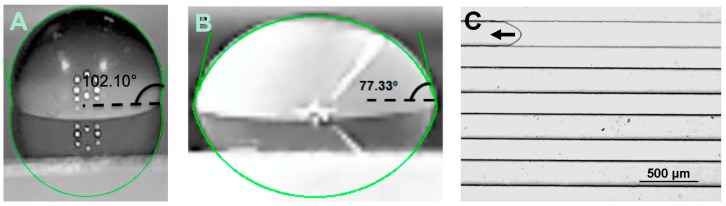
Representative images show the measured contact angles of human plasma on different surfaces. (**A**) Contact angle of 10 μL droplet of plasma on a surface of PDMS (control); (**B**) The static contact angle of a 10 μL droplet of plasma on Pluronic functionalized PDMS (4 µL/g); (**C**) The dynamic contact angle of plasma running through a resistor channel. The arrow indicates the flow direction.

**Figure 3:**
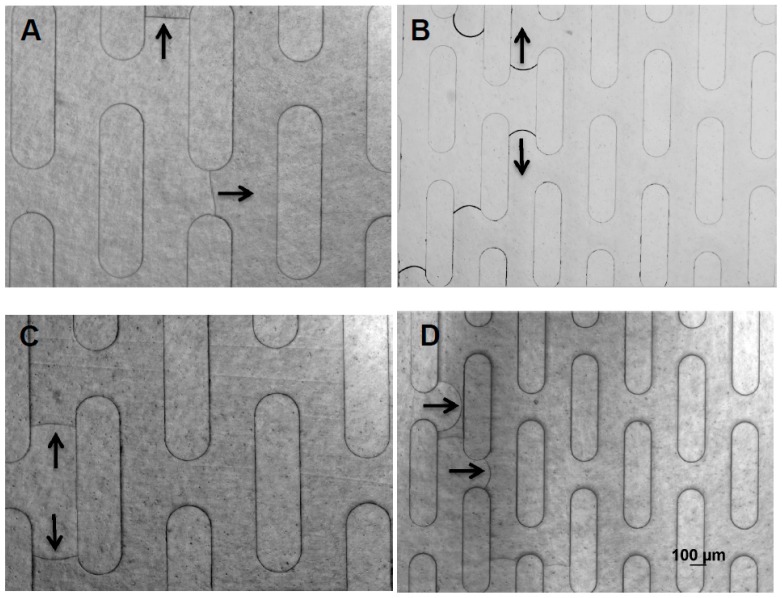
Representative results show the dynamic contact angles in the capillary pumps (CP). (**A**) CP1, Pluronic functionalized PDMS (Pluronic/PDMS (4 µL/g)); (**B**) CP2, Pluronic functionalized PDMS (Pluronic/PDMS (4 µL/g)); (**C**) CP1, Pluronic functionalized PDMS (Pluronic/PDMS (2 µL/g)); (**D**) CP2, Pluronic functionalized PDMS (Pluronic/PDMS (2 µL/g)). The black arrows indicated the flow direction in the capillary pumps.

Figure 2A,B show the representative images of static contact angles of 10 μL droplet of plasma on PDMS surface (control) and Pluronic functionalized PDMS surface, respectively. The dynamic contact angle was measured for sets of identical microchannels, with the only difference being the concentrations of Pluronic in PDMS. Figure 2C shows the dynamic contact angle of plasma running through a flow resistor channel. Table 3 shows the magnitude of the stabilized static contact angles after 20 min and measured dynamic contact angles. For the Pluronic functionalized samples, the difference in concentrations (2 µL/g and 4 µL/g) of Pluronic had a small impact on the static contact angle. On the other hand, the Pluronic concentration and channel resistance had a greater effect on the dynamic contact angle, as shown in Figure 2C and Table 3. Dynamic contact angles proved more hydrophilic with higher Pluronic concentration, and the flow resistance showed a major influence in both the magnitude of the contact angles, and more importantly, the flow rate, which was discussed in the following section. It is known that the pillar aspect ratio and their positioning in the capillary pumps, as well as the physical properties of the fluid (e.g., surface tension, viscosity), have a great impact on the capillary phenomena in the microchannel [10,35]. Contact angles were also examined in both the first and second capillary pumps. In a similar way to the flow resistor’s contact angles, the capillary pump’s contact angles had a tendency to decrease with higher concentrations of Pluronic. The effects of the resistance, determined by the microstructures in the capillary pump, and the flow rate, on the magnitude of the contact angle, can be observed in Figure 3. The contact angle proved to be more hydrophilic for the secondary capillary pump than those of the first capillary pump. The dynamic contact angles were usually lowest in magnitude for the secondary capillary pump, as the wetting velocity is decreased as a consequence of the additive resistance from all previous functional units (as these units are connected in series). 

### 3.2.Water Treatment

The surface properties of Pluronic functionalized PDMS were further enhanced by deionized (DI) water treatment, which can trigger gradient-induced migration of the Pluronic molecules embedded in the PDMS. This treatment would then result in enhancing the hydrophilic properties of the microfluidic channels, and it would also make the surface more stable and less prone to non-specific adsorption. The patterned side of the fluid layer, as well as the surface of the interfacing layer, were placed in contact with DI water for 24 h, before bonding both layers together. Contact angles were measured and proved to be much lower for the devices immersed in water compared to those not treated with water (Table 3), and the flow rate (discussed in the following section) was, as a consequence, higher for the water treated devices.

Both static and dynamic contact angles were measured to observe the effect of the water treatment. The water treated static contact angle only presented a very slight decrease in comparison of its non-treated counterpart, as shown in Table 3. However, the dynamic contact angles presented a more evident change, generally lower for the water treated devices, because this treatment triggers a gradient, leading the Pluronic molecules to merge to the surface of the microchannels. There were large variations in dynamic contact angles measurements for non-water treated devices, which may be mainly because of the non-uniform distributions of Pluronic molecules on the channel wall surfaces, leading to differences, device by device. 

### 3.3. Flow Rates

Liquid flow rates through the LFD can be regulated by modifying the hydrophilicity of the inner channel walls, and varying the dimensions of the microstructures and the spacing between them in the capillary pump, as well as the dimensions of the flow resistor, resulting in their resistance changes. These allow for providing control for the binding of proteins to antibodies, and further to enhance the detection. The CPs determine the filling front of the system, and their resistance is highest when they are almost full. However, the resistance of CPs is a few orders of magnitude smaller than the total flow resistance of the capillary system. In addition, the concentration of Pluronic also had to be optimized. In order to be able to predict the effect of the combination of the resistance and the Pluronic concentration on the flow speed, in situ dynamic contact angle measurements were performed, which were then compared with the time-lapse results. The concentration of Pluronic F127 and geometric flow resistances had great impact in the flow speed of the sample. Higher concentrations of Pluronic reduced the contact angle, therefore increasing the hydrophilicity of the device, and allowing for higher flow rates. 

Flow rate was highly consistent for the first CP due to the fact that a large portion consists of an open window where the plasma separation membrane was placed (Figure 1C). Table 4 shows the relationship between the flow rates in the secondary capillary waste pump, the geometry based fluidic resistance, and the concentrations of Pluronic. The flow rate in it had a general tendency to increase as resistance decreased, and as concentrations of Pluronic increased (Table 4). 

**Figure 4:**
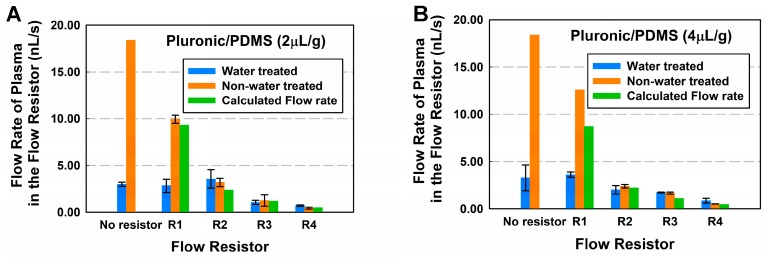
The flow rates of plasma in the flow resistor made of (**A**) Pluronic/PDMS (2 µL/g); (**B**) Pluronic/PDMS (4 µL/g).

Flow rate was also measured and compared for devices with and without the water treatment, for both the entire device and the flow resistor portion. Longer flow resistors encoded higher fluidic resistance, therefore reducing the magnitude of the flow rate. Flow rate was measured by measuring the time required for the fluid to flow through each component of the device. Figure 4A,B and Table 5 show the relationship between the flow rates of the flow resistor, the geometry based fluidic resistance, and the concentrations of Pluronic. The highest flow rate (18.4 nL/s) achieved was for devices with no flow resistor and without water treatment, and the lower flow rates were achieved with the longest flow resistor (0.43 nL/s) and a concentration of 2 μL/g. 

Time-lapse imaging using the Ti microscope is able to analyze how the flow rate decays over the length of the channel. Even though the flow rate decays over the length of the resistor, the flow rate of the device with lower fluidic resistance is higher at all times. The decay in speed occurs not only due to viscous dissipation forces, but predominately due to the increase in length of the filled length in the channel, and the friction due to the bulk viscosity [36]. In addition, for the water treated devices, even though contact angles proved to be more hydrophilic (Table 3), and flow rate was higher at the entrance of the channels, it was observed during experimentation that the effect of drying in channels was more evident in those devices, which resulted in the full length flow rate being lower compared to that of the non-treated ones.

**Figure 5:**
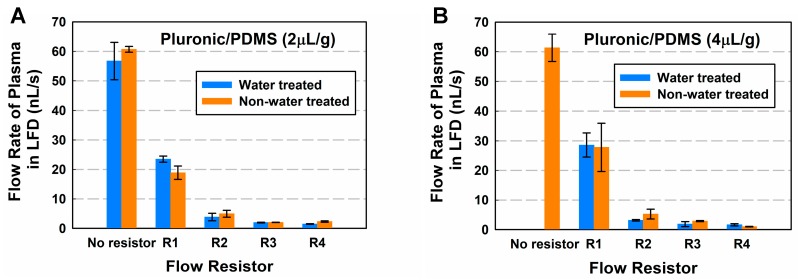
The flow rates of plasma in the entire LFD made of (**A**) Pluronic/PDMS (2 µL/g); (**B**) Pluronic/PDMS (4 µL/g).

Figure 5A,B and Table 6 show the effect of the resistance, the concentration of Pluronic and the water treatment on the flow rate of the entire device. The resistance has the most impact on the flow rate. 

Finally, the experimental results were validated using the equations in Section 2.3.1. Both the static and dynamic contact angles were evaluated separately to determine which one would be a better predictor of the flow rate. Results showed that the static contact angles could provide a good approximation to the experimental results, while the dynamic contact angles were a little more complex to use in the flow equations, because the contact angles (*α_l_* and *α_r_*) with two of the side microchannel walls remain unknown. Table 6 shows the calculated flow rates by assuming the four angles to be equivalent to the measured static contact angle. Results showed that static contact angle can serve as good predictor of the flow rate, even though it does not account for the resistance of the microchannels.

### 3.4. Plasma Separation

**Figure 6:**
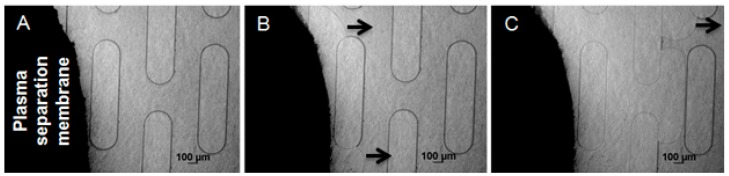
Plasma separation from whole blood. (**A**) Ready to separate; (**B**) Start to separate; (**C**) Continuous separation. The black arrows indicate the direction of the flow.

The 0.9 cm^2^ membrane can separate up to 36 µL of plasma from 45 µL of whole blood. Thirty microliters of whole blood (Innovative Research, Novi, MI, USA) was then deposited on top of the membrane, and slowly absorbed by it. Blood cells and platelets were trapped on the top part of the membrane, due to their sizes being larger than the membrane’s pores, and only plasma (24 µL) was able to pass through the membrane. Because of the hydrophilic surfaces of the microstructures in the capillary pump, and the close contact between the membrane and those microstructures, the plasma was continuously driven through the microchannels. High quality separated plasma (no leakage of red or white blood cells) was obtained with the GR membrane. Figure 6 shows the plasma separation process, beginning by pipetting human whole blood on top of the GR membrane. Table 7 compares the average velocity of the fluid at the entrance of the flow resistor for commercially available plasma (separated using other methods, such as centrifugation) and plasma separated using the Vivid GR separation membrane. The velocity of the separated plasma was highly consistent with that of the centrifuged plasma.

### 3.5. IgG Detection 

**Figure 7:**
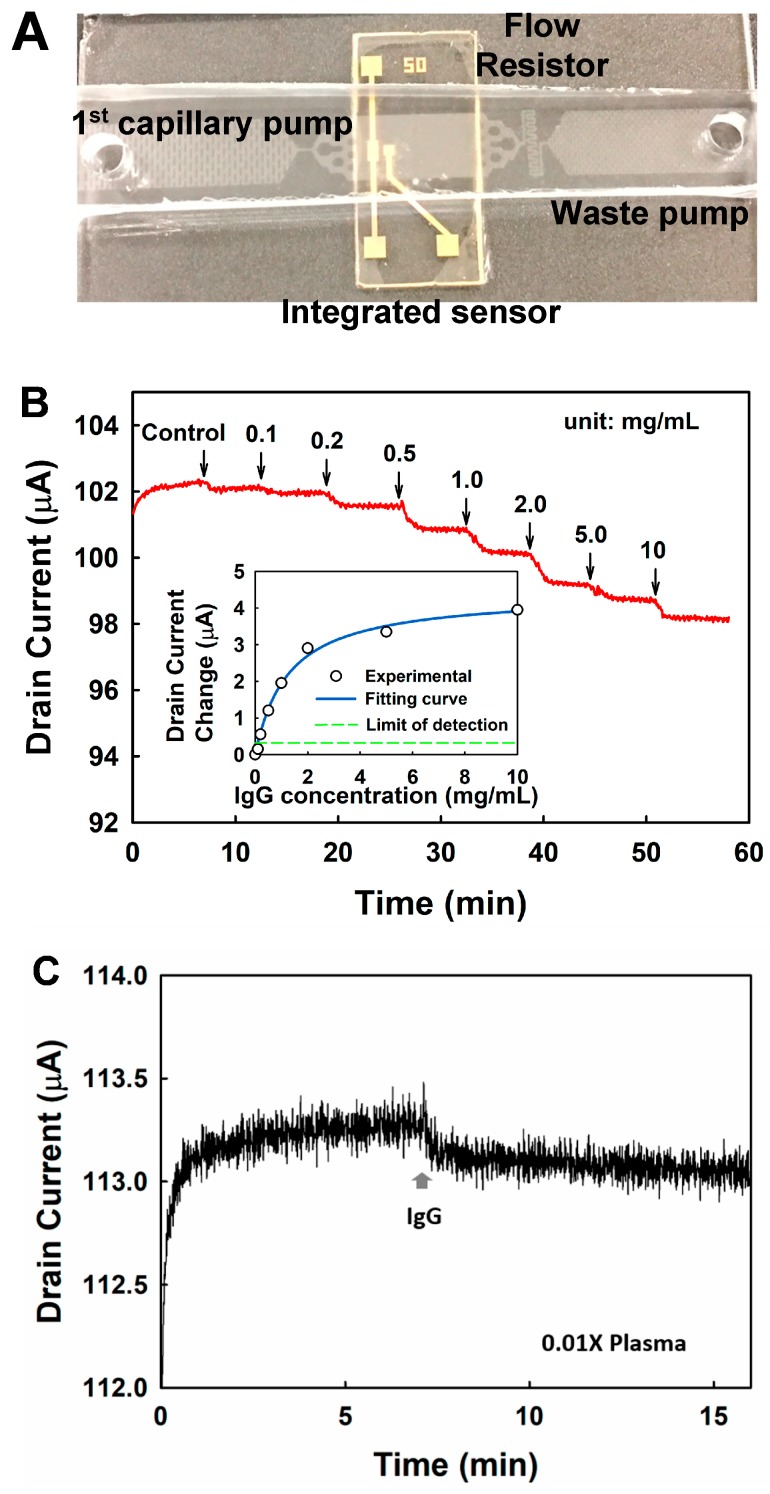
Real-time detection of IgG using nanoelectronic sensor integrated with the LFD. (**A**) A representative picture shows a fabricated LFD integrated with a nanoelectronic biosensor; (**B**) Continuous measurement of the drain current as responding to the additions of IgG with increasing concentrations; (**C**) The detection of IgG directly from human plasma.

To demonstrate biomolecule sensing using the integrated LFD, a nanoelectronic sensor was integrated with the LFD for the detection of immunoglobulins from buffer solution and human plasma (Figure 7A). Immunoglobulins are glycoprotein molecules produced by white blood cells, and they are a vital component of the immune system because they can recognize and bind to antigens, such as bacteria or viruses, and aid in their destruction. Immunoglobulins are specific to each type of foreign substance. For example, antibodies made in response to an infectious disease, such as tuberculosis, only bind to tuberculosis bacteria. IgG is the most abundant immunoglobulin in human plasma.

GFET biosensor operates by measuring the conductance change of the graphene channel induced by the specific adsorption of the target molecules on it. To test the performance of the GFET biosensors, 10 μL IgG (in 0.01× PBS) with different concentrations were injected into the LFD successively, and the drain current was monitored. Continuous decreasing of the drain current was observed upon the addition of the IgG with increasing concentrations, as shown in Figure 7B. This is because the specific binding of the positively charged IgG molecules can introduce *n*-type doping in graphene. As the major current carrier is whole when the gate voltage is stabilized at 0 V, the *n*-type doping effect reduces the number of the current carrier, and thus leads to the decrease of the drain current. The changes of the drain current with respect to the control are plotted in Figure 7B. The result can be well fitted with the Langmuir isotherm equation:

(9) $$\Delta I_{d}=\frac{\Delta I_{d}^{\max}\times \left[\mathrm{IgG}\right]}{\left[\mathrm{IgG}\right]+K_{d}}$$

in which $\Delta I_{d}^{\max}$ is the response of the biosensor when the binding sites are saturated, and $K_{d}$ is the dissociation constant of the aptamer–IgG complex. The fitting yields $\Delta I_{d}^{\max}$ as 4.40 μA, and $K_{d}$ as 1.26 mg/mL, with a standard error as 0.07 μΑ. Based on the calibration curve from fitting, as shown in the insert figure of Figure 7B, the limit of detection is estimated to be 0.12 mg/mL with a signal-to-noise ratio of 3. The sensitivity is around 2 μΑ·mL/mg in the linear detection range (0.12–1.0 mg/mL).

IgG was detected directly from plasma using the LFD integrated with the GFET. Ten microliters of 0.01× PBS was first loaded in the device, followed by 10 μL plasma sample that was diluted by a factor of 100. The drain current was monitored continuously. As shown in Figure 7C, the drain current decreases by around 0.18 μA upon the addition of plasma. The concentration of the IgG was estimated to be 0.09 mg/mL in 0.01× plasma, based on the calibration curve as shown in the insert figure of Figure 7B, corresponding to 9 mg/mL in undiluted plasma, which is within the normal range in human blood [37]. The result indicates that the developed LFD has the flexibility to integrate on-chip sensors, and shows a great potential to be used for biomarker detection from complex fluid. Further studies on improving GFET sensitivity and target selectivity are under investigation, and the results will be reported separately. 

## 4. Conclusions

In order to bridge the healthcare gap between developing and developed countries, there is a growing need for integrated, low cost solutions that are easy to handle, and that are able to provide high quality results. Microfluidics is a powerful alternative for addressing this objective, however, most of current technologies are not fully integrated in-chip, and therefore require external equipment which is expensive and not portable. The LFD developed in this work shows potential to overcome this problematic, by integrating all functions in-chip, and allowing for the performance of entire laboratory protocols for immunoassays without the need of any external equipment. Detailed work was performed, to investigate the controlling of flow rates in the LFD by manipulating channel surface properties and geometrical features of the device, including static and dynamic contact angles, functionalization of PDMS, fluidic resistances, and microstructures in capillary pumps. The LFD combines the ability to separate plasma from a small sample of whole blood, initiate and control the fluid flow by taking advantage of capillary forces, with the potential to implement a wide range of immunoassays. Results from this work could provide a more comprehensive understanding of the fluid flow and function integration using a LFD. 

PDMS was functionalized using Pluronic F127, in order to render it hydrophilic. Because time is a critical factor in performing biomolecules’ binding and detection, flow rate was controlled by several factors, including the microstructures’ sizes and positions in the capillary pumps, concentrations of Pluronic, and by tuning the resistance of the flow resistor. Varied plasma flow rates were achieved in the whole device. Even though water treatment presents an alternative for stabilizing Pluronic molecules on the PDMS surface and preventing non-specific absorption, it is important to find ways to control and prevent drying due to evaporation. 

The current platform is designed for the detection of a single biomarker, but it will be further modified to obtain multiplexing capabilities. In addition, to develop the LFD into a more reliable and accurate platform, further studies need to focus on investigation of improving nanoelectronic sensor’s sensitivity and target selectivity, and testing different types of biosensors, including colorimetric and fluorescence sensors, optimizing flow rate control in regarding to the specific targeted biomarkers and integrated sensors, etc. These would make this platform sensitive, versatile, and easy to use, and allow generation of more meaningful and conclusive information for clinical diagnosis. 

## Figures and Tables

**Table 1:** List of integrated flow resistors with different flow-rate resistances and hydraulic resistances and corresponding flow resistances of LFD.

Flow Resistors	Flow-Rate Resistance (m^−3^)	Hydraulic Resistance for Plasma (kg/m^4^·s)	Flow Resistance of LFD (m^−3^)
R1	1.02 × 10^17^	1.69 × 10^14^	1.49 × 10^17^
R2	4.35 × 10^17^	7.22 × 10^14^	4.94 × 10^17^
R3	8.80 × 10^17^	1.46 × 10^15^	9.40 × 10^17^
R4	2.35 × 10^18^	3.90 × 10^15^	2.44 × 10^18^

**Table 2:** Summary of measured parameters for characterization of flow rates.

Characterization of Flow Rates			Plasma	Contact Angles
Characterized Parameters	Pluronic Functionalized polydimethylsiloxane (PDMS)	Non-water treated	√	Static
			√	Dynamic
		Water treated	√	Static
			√	Dynamic
		No flow resistor	√	-
		R1	√	-
		R2	√	-
		R3	√	-
		R4	√	-
	Capillary Pump (CP)	Waste CP	√	-
	Lateral Flow Device		√	-

**Table 3:** Measured static and dynamic contact angles of plasma.

**Static Contact Angles for Plasma (Mean ± SD)**				
Control PDMS	**Non-Water Treated**		**Water Treated**	
	Pluronic/PDMS (2 µL/g)	Pluronic/PDMS (4 µL/g)	Pluronic/PDMS (2 µL/g)	Pluronic/PDMS (4 µL/g)
101.95° ± 1.68°	68.42° ± 5.22°	69.85° ± 1.30°	65.60° ± 2.67°	69.74° ± 1.65°
**Dynamic Contact Angles for Plasma (Mean ± SD)**				
Flow Resistors	**Non-Water Treated**		**Water Treated**	
	Pluronic/PDMS (2 µL/g)	Pluronic/PDMS (4 µL/g)	Pluronic/PDMS (2 µL/g)	Pluronic/PDMS (4 µL/g)
R1	106.80° ± 4.66°	73.58° ± 15.67°	57.25° ± 4.86°	68.21° ± 3.87°
R2	104.00° ± 1.00°	58.90° ± 4.96°	54.89° ± 5.92°	67.00° ± 9.53°
R3	108.26° ± 3.31°	95.45° ± 2.85°	44.30° ± 6.29°	45.46° ± 3.60°
R4	94.00° ± 3.67°	91.30° ± 1.67°	38.00° ± 6.68°	44.00° ± 9.11°

**Table 4:** Flow rates of plasma in the waste capillary pump.

Flow Rate of Waste Pump (nL/s) (Mean ± SD)		
Flow Resistors	Pluronic/PDMS (2 µL/g)	Pluronic/PDMS (4 µL/g)
No resistor	38.55 ± 7.85	34.61 ± 1.58
R1	8.88 ± 1.80	12.11 ± 3.72
R2	2.08 ± 0.52	1.43 ± 0.46
R3	0.90 ± 0.05	1.34 ± 0.06
R4	0.32 ± 0.03	0.61 ± 0.11

**Table 5:** Flow rates of plasma in the flow resistors.

Flow Rate of Plasma in the Flow Resistor (nL/s) (Mean ± SD) (Experiment)					Calculated Flow Rate	
Flow Resistors	Water Treated		Non-Water Treated			
	Pluronic/PDMS (2 µL/g)	Pluronic/PDMS (4 µL/g)	Pluronic/PDMS (2 µL/g)	Pluronic/PDMS (4 µL/g)	Pluronic/PDMS (2 µL/g)	Pluronic/PDMS (4 µL/g)
No resistor	2.96 ± 0.22	3.27 ± 1.37	18.4 ± 0.00	18.40 ± 0.00	-	-
R1	2.82 ± 0.72	3.61 ± 0.28	9.94 ± 0.43	12.60 ± 0.00	9.31	8.72
R2	3.55 ± 1.00	1.99 ± 0.45	3.17 ± 0.46	2.37 ± 0.18	2.35	2.20
R3	1.04 ± 0.22	1.72 ± 0.04	1.24 ± 0.61	1.65 ± 0.11	1.17	1.09
R4	0.70 ± 0.08	0.86 ± 0.25	0.43 ± 0.10	0.52 ± 0.01	0.49	0.46

**Table 6:** Flow rates of plasma in the LFD.

Flow Rate of Plasma in LFD (nL/s) (Mean ± SD)				
Flow Resistors	Water Treated		Non-Water Treated	
	Pluronic/PDMS (2 µL/g)	Pluronic/PDMS (4 µL/g)	Pluronic/PDMS (2 µL/g)	Pluronic/PDMS (4 µL/g)
No resistor	56.72 ± 6.35	-	60.70 ± 0.98	61.30 ± 4.62
R1	23.49 ± 1.04	28.57 ± 4.10	18.88 ± 2.27	27.76 ± 8.16
R2	3.84 ± 1.27	3.13 ± 0.24	4.96 ± 1.17	5.23 ± 1.66
R3	1.96 ± 0.12	1.85 ± 0.84	2.02 ± 0.05	2.86 ± 0.19
R4	1.48 ± 0.09	1.62 ± 0.38	2.32 ± 0.22	0.99 ± 0.09

**Table 7:** Comparison of flow rates of the flow resistor (entrance) for separated plasma from whole blood and centrifuged plasma. The substrate was Pluronic functionalized PDMS (Pluronic/PDMS (4 µL/g)).

**The LFD with Flow Resistor R3**	**Fluid Velocity and Rate**	**Plasma**	**Separated Plasma from Whole Blood**
	Average Velocity (Mean ± SD) (µm/s)	375 ± 73.54	385 ± 24.09
	Flow Rate (nL/s)	1.87 ± 0.36	1.92 ± 0.12
